# Medical genetics and genomic medicine in the Dominican Republic: challenges and opportunities

**DOI:** 10.1002/mgg3.224

**Published:** 2016-05-12

**Authors:** Juvianee I. Estrada‐Veras, Giselle A. Cabrera‐Peña, Ceila Pérez‐Estrella de Ferrán

**Affiliations:** ^1^ Medical Genetics BranchNational Human Genome Research InstituteSection of Human Biochemical GeneticsNational Institutes of HealthBethesdaMaryland; ^2^ Primary Care UnitCarol Morgan School of Santo DomingoSanto DomingoDominican Republic; ^3^ Department of PediatricsRobert Reid Cabral Children's HospitalSanto DomingoDominican Republic

## Abstract

Medical genetics and genomic medicine in the Dominican Republic: challenges and opportunities.

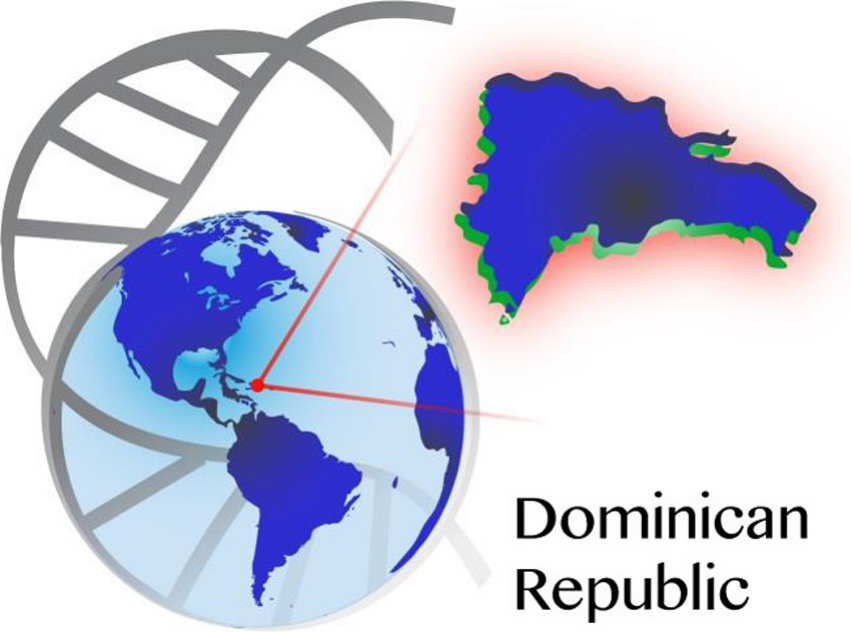

## Introduction

“There is a country in the world situated right in the sun's path. A native of the night. Situated in an improbable archipelago of sugar and alcohol.” These are the words used by the Dominican national poet, Mir (1949), to describe his beloved Dominican Republic (DR). A country in the Spanish Caribbean with mixed Spanish, African, and native Caribbean Indian ancestries whose culture and economy have been tightly linked to sugar and rum. The capital city, Santo Domingo de Guzman, is currently the largest city of the Central American and Caribbean regions in terms of population. Until 1492, the island of Quisqueya, today called the island of Hispaniola, was populated by the Arawaks (Amerindians). After Christopher Columbus' first voyage to the new world in 1492, Europeans and Africans settled in the island. Throughout the centuries, waves of immigrations have contributed to the modern Dominican population (Pan American Health Organization 2012; Agency, C. I. A. 2013; World Health Organization 2013).

**Figure 1:**
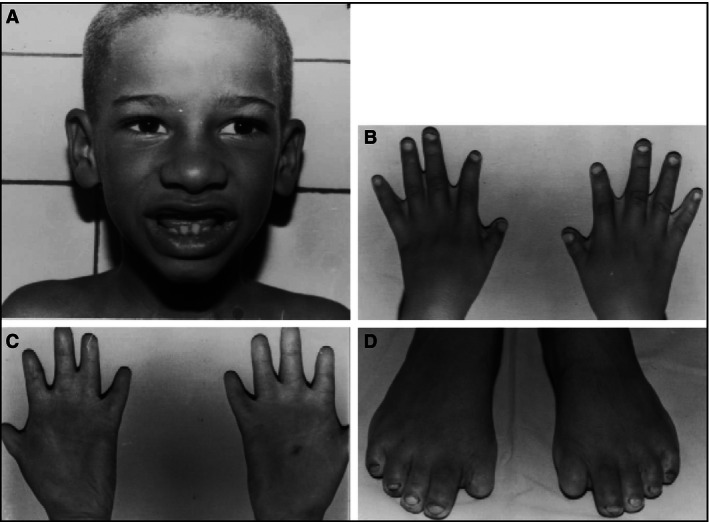
Dominican child with odonto‐tricho‐ungual‐digital‐palmar syndrome. (A) Note discolored hair and incomplete dentition. (B) Interdigital folds and brachydactyly of thumbs. (C) Brachydactyly with transverse palmar creases. (D) Absence of the first nails toe and dystrophy of the other nails. (Taken from Mendoza 1997).

The fields of genomic medicine and clinical genetics are not entirely unknown in the DR, however, they are not independent fields and the practice of these specialties is limited. Throughout the history of medicine in the DR, genetic cases have been mentioned. In 1533, the first autopsy in the new world was performed on twin sisters who were joined in the back (Jimenez 1978). Since then and until today, genetic cases have been present in the island of Hispaniola. Dr. Hugo Mendoza, a Dominican pediatrician with a strong interest in rare and genetic diseases, was the pioneer of genetics in the DR. Without formal training in the field, he built the foundation of what is today the largest genetic service in Santo Domingo. Thanks to his efforts, cases of alkaptonuria (Goicochea de Jorge et al. 2002) were reported in the literature, as well as a new form of ectodermal dysplasia, the odonto‐tricho‐ungual‐digital‐palmar syndrome (Mendoza 1997); (see Fig. 1).

Dr. Teofilo Gautier, a Dominican physician, contributed to the field of genetics by reporting the incidence as well as the characteristics of 5‐alpha reductase in the DR (Imperato‐McGinley et al. 1974). These cases were of interest due to its prevalence in the small remote village of Las Salinas, where 12 out of 13 families had one or more male family members who carried the genetic mutation, though not all the carriers of the mutation were affected. The overall incidence rate for the village was, 1 in every 90 males were affected carriers.

The field of genetics is still underrepresented and the need is evident. Today, a group of pediatricians and other health care workers are introducing the field of genetics and raising awareness for children and adults with rare and genetic diseases. The Dominican government is also paying attention to this field of medicine.

## Dominican Republic: General Statistics

**Figure 2:**
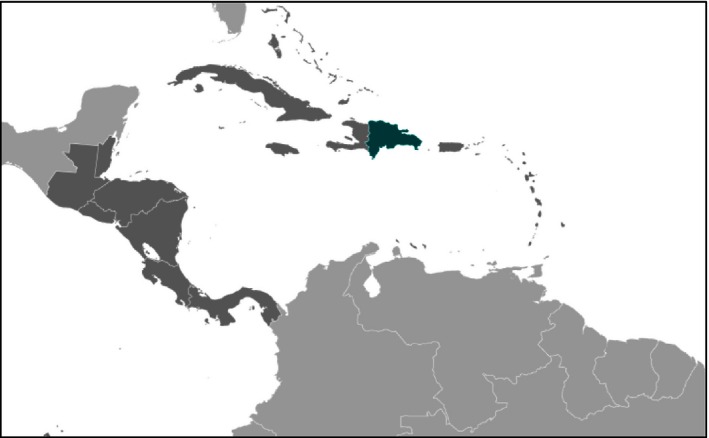
The Dominican Republic in the Caribbean.

**Figure 3:**
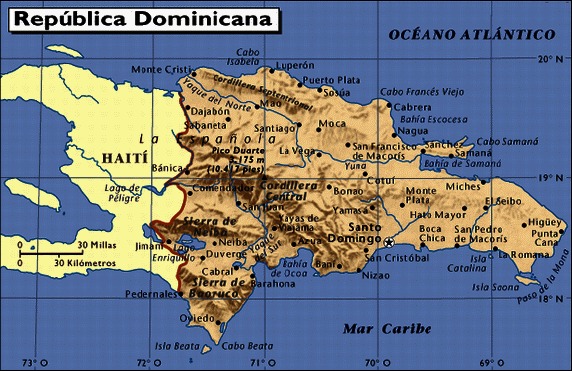
The political division of the Dominican Republic.

The DR is a country of 48,442 km^2^ or 18,704 miles^2^, roughly the combined sizes of the states of New Hampshire and Vermont. The country is politically divided into 31 provinces and Santo Domingo de Guzman is located in the National District. As part of the Greater Antilles of the Caribbean, it shares the island of Hispaniola with the Republic of Haiti as its western neighbor. The country borders on the north with the Atlantic Ocean and with the Caribbean Sea on the south. The Mona Passage, to the east, separates the DR from the Island of Puerto Rico (Figs. 2, 3). The estimated population for the DR in 2015 was just below 10.5 million with a large percentage of the population living in cities such as Santo Domingo and Santiago de los Caballeros, (See Table 1). An estimated 1–1.5 million Dominicans live outside the country. The population is mainly composed of different ethnicities, such as blacks (11%), mulattoes (73%), and whites (16%). This is the gross composition since the Dominican population is described as being the most diverse and mixed in terms of ethnicities among the Caribbean islands. Tracing the exact ethnic composition in the DR is challenging secondary to the lack of population studies, proper documentation and statistics as well as a population bias in regard to ethnic identity. However, it is strongly accepted that Dominicans are not purely blacks, mulattoes, or whites, but the perfect blending of ethnicities (Oficina Nacional de Estadistica (ONE) 2010a,c; Pan American Health Organization 2012; Agency, C. I. A. 2013).

**Table 1:** General characteristics of the Dominican Republic

Capital city	Santo Domingo de Guzman
Nationality	Dominican
Political division	31 provinces
Government	Constitutional Democracy‐Republic
Ethnic groups	Mulattoes or mixed = 73%, White = 16%, Black = 11%
Official language	Spanish – Castilian
Religion	Roman Catholic = 95%, Others = 5%
Climate	Tropical
Population	10, 478, 756 (estimate July 2015)
Population growth rate	1.23% (estimate for 2015)
Area	48,442 km^2^ or 18,704 miles^2^
Population density	216.3/km^2^ or 560.2/mile^2^
Urban population	79% of total population
GDP (purchasing power parity)	$US 138.5 billion in 2014

Once an agricultural powerhouse, the DR is the largest economy (according to the U.S. State Department) in the Caribbean and Central American region. It is an upper middle‐income developing country with an inflation rate of 3%. Today, the economy of the country relies on the service sector. The economy's largest employers are now in the telecommunications, tourism, and free trade zones. The economy is highly dependent upon the US, the destination for approximately half of exports. Remittances from the US amount to about 7% of GDP (US3.4 billion in 2010) which is equivalent to about a third of exports and two‐thirds of tourism receipts. In 2015, the country was the most visited destination in the Caribbean. An undetermined percentage of visitors relocate in the DR and most of the times start a family with locals adding their ethnicity to the Dominican melting pot. The Gross Domestic Product (GDP‐PPP) of the DR in 2014 was US 138.5 billion with 2.3% spent on healthcare and 3.8% spent on education. There is strong lobbying by the Dominican population and leadership sectors for an increase in resources for healthcare and education. An increase in the allocations for healthcare and education of 5% and 4% of the GDP is being requested (Oficina Nacional de Estadistica 2010b; Agency, C. I. A. 2013). The country suffers from marked income inequality.

## Population Diversity and Immigrations to the Dominican Republic

The Taino, an Arawak subgroup, were the first Caribbean natives encountered by Christopher Columbus on Hispaniola in 1492 (Agency, C. I. A. 2013). They originated from the Orinoco and Amazon rivers area in South America. The Tainos succumbed to diseases introduced by Europeans to which they had no immunity and to torture by the new settlers. Christopher Columbus explored and claimed the island on his first voyage and it became the headquarters for the Spanish conquest of the Caribbean and the American mainland. In 1697, Spain recognized French dominion over the western third of the island, which in 1804 became the Republic of Haiti. Spanish Santo Domingo sought to gain its own independence in 1821 but was conquered and ruled by the Haitians for 22 years; it finally gained independence as the DR in 1844. In 1861, the Dominicans voluntarily returned to the Spanish Empire, but they restored independence in 1865. These events had significant influence in establishing the modern Dominican population makeup. Africans, Caribbean Indians, and European settlers, especially from Spain and France provided the foundation for the modern and ethnically heterogeneous Dominican population. Sephardic Jews who were exiled from Spain and the Mediterranean area between 1492 and 1497 and those who migrated in the 1700s also contribute to the Dominican ethnic ancestry (Oficina Nacional de Estadistica 2010c; Pan American Health Organization 2012; Agency, C. I. A. 2013).

In modern days and according to genealogical DNA testing, the genetic makeup of the Dominican population is estimated to be 52% European, 40% Sub‐Saharan African, and 8% Native American‐Taino (Montinaro et al. 2015). The general population of the country is divided into three ethno‐racial groups. Mulattoes or mixed race Dominicans are 73% of the population. They are mainly of African and European descent. White Europeans of mainly Spanish and French descent compose 16% of the population and Black Africans compose the remaining 11%. Other groups include descendants of Lebanese, Syrians, and Palestinians. During the Second World War another wave of Sephardic Jews migrants, believed to be up to 100 thousand, relocated in the northern coast of the DR. A large number later immigrated to the United States, but some are still present in the DR. The exact number of Dominicans with Jewish lineages is not known due to the inter‐mixing between Jews and Dominicans over a period of more than five centuries and to the fact that many Jews converted to Roman Catholicism to avoid religious persecution (Oficina Nacional de Estadistica 2010c; Agency, C. I. A. 2013; Montinaro et al. 2015).

In the twentieth century, Chinese, Lebanese, Syrians, Japanese, and Koreans migrated to the country to work in agriculture and trade. The Chinese population in the DR is estimated to be around 50, 000. The Arab community is increasing. There are descendants of immigrants who came from other Caribbean islands especially from the British Caribbean to work in sugar cane fields. More recently, immigration from Europe and the United States, the “expats”, is at an all‐time high. The recent European migration is composed mainly by Italians, French, British, and Germans. In 2012, the number of people with foreign origin was 768,783 according to a National Statistics Office. Of these, 524,632 were born outside of the DR (see Table 2) (Oficina Nacional de Estadistica 2010c).

**Table 2:** Population of foreign origin in the Dominican Republic in 2012

Country of origin	Number living in the Dominican Republic
Republic of Haiti	458,233
United States (excluding PR)	13,514
Spain	6720
Puerto Rico	4416 and rising
Italy	4044
China	3643
France	3599
Venezuela	3434
Cuba	3145
Colombia	2738
Germany	1792

## Healthcare in the Dominican Republic

### Health determinants

As it applies to worldwide health, health determinants in the DR are a combination of many factors that together affect the health of individuals and communities in different ways. Health determinants include social and economic environment, physical environment, and the person's individual characteristics, genetics, and behaviors. In the case of the DR, a country that has seen population and urban growth with little to no planning, social and economic environments are the determinants with the most impact on the health of the population. The greater the gap between the richest and the poorest in a community, the greater is the difference in health and access to health services. Also, low education levels are linked with poor health, more stress, and lower self‐confidence (Pan American Health Organization 2012). In 2004, poverty indicators in the country reached their highest levels in two decades. Extreme poverty was estimated at 15.9% and general poverty at 43%. By 2010, extreme poverty declined to 10.4%, making it unlikely that the country will reach the Millennium Development Goal of reducing extreme poverty to 5.4% by 2015. The poorer population's share of the country's income is extremely low and a more equitable distribution is not happening anytime soon. In the last decades, unemployment has averaged 16.4%. Illiteracy in 2007 was calculated at 10.7% (Oficina Nacional de Estadistica 2010b; Pan American Health Organization 2012; Agency, C. I. A. 2013; The World Bank 2014).

Access to clean water and sanitation has improved in the recent years with 84.7% of the population having access to a water source and 84% having access to sanitation facilities. Access to healthcare in the DR is complex as in most countries of the developing world. Healthcare is divided between the private and public sector with the vast majority of the population relying on public health services. 2011 estimates report that the physician density in the country was 1.49:1000 and hospital bed density was 1.7:1000. All of these factors contribute to the difficult situation that the Dominican health system is undergoing which affects mainly children who live below the poverty line. In 2013, 4% of the children under the age of five were underweight and vector‐borne illness such as dengue and malaria, and more recently, Chikungunya and Zika viruses, have been challenging for an already struggling healthcare system. Bacterial diarrhea, typhoid fever, and malnutrition are still public health issues among the pediatric population. Upper respiratory infections and other respiratory illnesses are the most common cause for which mothers seek medical attention for their children. To these determinants, the consequences of natural disasters such as hurricanes have not been included. The contribution of genetics in this scenario is not known (Oficina Nacional de Estadistica 2010b, 2007‐2012, 2008–2013a; Pan American Health Organization 2012).

### The health system in the Dominican Republic

The health system in the DR is comprised of a public sector and a private sector. The government run public sector consists of the Ministry of Public Health and Social Welfare, the National Health Council, the Social Security Treasury, and the National Health Insurance program, which is the main public insurer. The private sector includes health risk administrators or private health insurance providers; private health services providers, and nongovernmental organizations (NGOs) (Pan American Health Organization 2012). There is an immense gap between the services provided by both sectors. Public health hospitals are commonly overwhelmed by the large number of patients and minimal resources. These hospitals are also understaffed and the medical community of the country goes on strike frequently since they consider the system to be broken, understaffed, overworked, and underpaid. The private sector on the other hand can provide services similar as those provided in the United States of America. A large number of Dominican physicians working in the private sector have been trained in residency and fellowship programs in the United States and Europe. These services are not available to everyone since “out‐of‐pocket” cost is elevated and insurance reimbursement is not accepted by some providers. The gap between the public and private health systems is such, that even though the DR is still battling basic public health issues, the country is being promoted internationally as a medical tourism destination and a plastic surgery Mecca in the Caribbean.

The Ministry of Public Health network has 1853 facilities composed of 1703 primary care units and 150 specialized second‐ and third‐level treatment centers. These specialized centers consist of 15 specialized hospitals, 11 regional hospitals, 20 provincial hospitals, and 104 municipal hospitals (Oficina Nacional de Estadistica 2007‐2012.

General Health Law 42–10, which was approved in 2001, has separated the functions of care delivery, leadership, and financing (Ministerio de Salud de la Republica Dominicana 2001). The nine regional health services are the health care service providers for the public, connected by a network of complexity levels with the capacity to provide at least minimum care as described in the Basic Health Plan. This plan however, does not address genetic or rare diseases. A quality assurance committee was created in 2009. This committee is in charge of overseeing the quality of the care being delivered as well as discusses and recommends proposals for policies and laws on various issues. Health care reform has been in development since 2008 and various health programs have been expanded and improved. New programs for chronic and mental diseases have been created, however, there is little to no mention on genetic and rare diseases that are chronic or that impact mental health. Until 2012, the new care models had not been reviewed or fully implemented. An important factor affecting this reform is that the human resources with the needed skills for proper implementation of this reform is limited or is lacking for certain areas.

In 2010, the public health sector had 56,240 employees. The physician density in 2011 was 1.49:1000 and the hospital bed density was 1.7:1000. The country's geographical distribution of doctors and nurses is unequal. The majority of healthcare professionals are concentrated in the cities with greater development. In the capital city, the physician density is 37.1:10,000 while in a small city such as La Romana, the physician density is 8.3:10,000. By 2012, there were 17,869 physicians, 15,748 nurses, and 466 psychologists registered with the health ministry for a country of more than 10 million inhabitants without taking into consideration the Haitian population that enters the DR only for medical care and returns to Haiti afterward. The differences between the regions and cities impair access, equity, and efficiency of health care (Oficina Nacional de Estadistica 2007‐2012, 2008–2013a; Pan American Health Organization 2012).

### Morbidity and mortality

The health information system uses the epidemiological surveillance, statistics of service, and vital statistics service to gather the country's epidemiological information. The epidemiological surveillance system is the only one with national coverage. The other two systems are characterized by fragmentation and poor consistency, and they provide little opportunity to apply data. These inadequacies result in the underreporting of much needed health information. Data of the Ministry of Health shows that a total of 6.9 million consults were reported in the public health registry in the year 2013. Of these consults, 713,000 were for new general medicine complaints, 629,000 were for new consult in pediatrics, 125,000 were new consults in the field of internal medicine, and 262,000 were obstetric new consults. There is little documentation of the specific medical problems that triggered these consults and how many of these were secondary to rare or a genetic disease is unknown (Oficina Nacional de Estadistica 2007‐2012, 2008–2013a; Pan American Health Organization 2012).

The most common cause of morbidity in the DR is communicable diseases, especially respiratory infections and vector‐borne diseases such as dengue fever and malaria. Dengue fever is endemic to the DR. In 2010, there were 12,166 cases reported with 49 cases succumbing to the disease. Malaria is also endemic to the DR. The number of malaria cases has been decreasing through the years. In 2010, there were 1643 cases detected (Pan American Health Organization 2012). Vaccine‐preventable diseases are still a public health problem in the DR, but there are successes; thanks to the national immunization program. However, there is still room for improvement. The transmission of wild poliovirus was interrupted in the DR in 1986, though there was a small vaccine‐related outbreak in the mountain region of the DR in the early 2000s. Reported cases for measles and rubella were nearly zero in 2001 and 2006, respectively. National MMR vaccine coverage in 2010 was 97% among people between the ages of 9 and 39 years. Neonatal tetanus is no longer a public health problem, but cases of tetanus in children, though rare, are still reported. This also applies to diphtheria. In 2013, the Pan American Health Organization declared the DR free of measles, mumps, and polio. In 2015, Latin America was declared free of these three conditions. Zoonoses such as rabies and leptospirosis are still reported in the country. Leprosy and parasitosis are a common cause of morbidity and public health efforts to reduce the incidence of these entities are underway. Migration, especially from Haiti, increases the incidence of these communicable diseases (Oficina Nacional de Estadistica 2008–2013a; Pan American Health Organization 2012).

In 2010, it was estimated that 48,550 people were living with HIV/AIDS and the prevalence in pregnant women was over 1%. An estimated 442 children were infected with HIV. More than half of the HIV‐positive pregnant women do not receive anti‐retroviral therapy. No cases of cholera were reported in the island of Hispaniola for more than 100 years until October of 2010, when an outbreak in Haiti was confirmed. The first case of cholera in the DR was detected in November 2010 when the presence of Vibrio cholerae O1 Ogawa serotype was confirmed. By June 2011 there were 10,760 cases reported resulting in 153 deaths. The case‐fatality rate was 1.4%. With all these frequent basic healthcare issues, one can question whether the DR health system has room to take care of rare and genetic diseases (Oficina Nacional de Estadistica 2008–2013a; Pan American Health Organization 2012).

In 2009, the Ministry of Public Health established the National Chronic, Non‐communicable Disease Prevention and Control Program. The program's goal is to promote health and help prevent and control chronic, noncommunicable diseases and their risk factors, and also to compile reliable data that will provide information on the actual presence and severity of these diseases within the Dominican population. An information system to help assess the extent of these diseases has not yet been developed. Among the diseases being targeted under this program are cardiovascular diseases, malignant neoplasm, diabetes, obesity, malnutrition, anemia, and mental disorders. Other public health problems of social nature such as domestic and gun violence are also included in this program. Interestingly, there is no distinction or discussion about a possible genetic cause for these primary care issues or whether any of the mental health issues could be the result of an undiagnosed rare disease or inborn error of metabolism. In the year 2013, there were a total of 708,000 people in the DR diagnosed with disabilities that are not specified in the national statistics (Oficina Nacional de Estadistica 2013).

In 2007, there were 22, 699 reported deaths in the DR. A total of 10,901 had a reported cause of death and 9822 had “other causes” as cause of death. Of those that had a known cause of death, many had an unspecified diagnosis or cause of death. For example, 785 cases were reported as other unspecified causes of death, 447 had unspecified stroke as causes of death with no differentiation between ischemic, hemorrhagic, or metabolic stroke. A total of 1976 cases had no cause of death reported. Death due to genetic disease has not been reported (Ministerio de Salud Publica de la Republica Dominicana 2007).

### Birth rate and maternal, infant, and childhood mortality

According to information given by the National Office of Statistics, in the year 2013, there were a total of 129,319 births in the DR. A total of 3312 infant deaths were reported that year for an infant mortality rate of 25.6:1000. For 2013, the fertility rate was reported at 2.5. Twenty‐six thousand abortions or miscarriages were reported during the same year. In the 2013 document, the number of stillbirths and premature babies are not mentioned (Oficina Nacional de Estadistica 2008–2013a). Maternal mortality was reported as 183:100,000 (Oficina Nacional de Estadistica 2008–2013b). This number is an estimate, since underreporting of deaths is frequent. In 2009, mortality in children under 5 years of age was 32:1000 live births. No downward trend in infant mortality has been observed in the last decade. Underreporting of infant death is also high and in 2010, it was estimated at 43.8%. The two leading causes of death in infants and children under the age of 5 years are septicemia and pneumonia (Pan American Health Organization 2012). Table 3 shows the population growth and reproductive indicators estimates for the year 2015.

**Table 3:** Population growth – reproductive estimates for 2015 for the Dominican Republic

Population indicator	2015 Estimate
Growth rate	1.23%
Birth rate	18.7 births: 1000
Death rate	4.55 deaths: 1000
Life expectancy at birth	77.9 years
Total fertility rate	2.33 children/woman
Maternal mortality rate	92 deaths: 100,000 live births
Infant mortality rate	18.8 deaths: 1000 live births
Contraceptive prevalence rate	73% in 2010

### Medical genetics, genetic counseling, and genetic testing services

**Figure 4:**
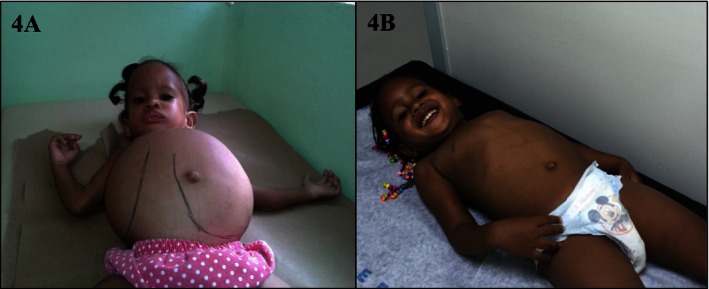
Dominican child with Gaucher disease. (A) Hepatosplenomegaly in a Dominican child with Gaucher disease prior to enzyme replacement therapy. (B) Regression of hepatosplenomegaly while on therapy with enzyme replacement therapy. (Courtesy of Dr. Ceila Pérez‐Estrella de Ferran, Robert Reid Cabral Children's Hospital.)

Medical genetics and the concept of rare and neglected disease are not unknown to the DR. For more than 20 years, there have been healthcare professionals with interest in this field of medicine and have been promoting the field and offering services to this patient population that is currently underrepresented within the Dominican health system. Of the 1853 health facilities of the National Health Ministry, only one has a dedicated clinical genetics service, The Robert Reid Cabral (RCC) Children's Hospital in Santo Domingo (Oficina Nacional de Estadistica 2007‐2012. This is the only public service medical genetics clinic that provides services to the entire country including cases that are referred from Haiti. This clinic is staffed by two pediatricians with more than 20 years of experience, but no formal training in genetics. This hospital also offers a diagnostic clinic established in 1976 where most of the genetic diseases are followed, though many cases remain undiagnosed secondary to limited resources. There is an inborn error of metabolism unit in this clinic as well. Cases of trisomy 21, Turner syndrome, cleft lip and palate, and other disorders are followed. The genetics clinic does not have statistics available that would give details on the frequency of diagnosis. In the inborn errors of metabolism clinic, even though the statistics are also poor, the unit has diagnosed and follows cases of mucopolysaccharidosis, Gaucher disease, Glycogen storage disease type I and II, Niemann–Pick disease, cystinosis, maple syrup urine disease, alkaptonuria, Fructosuria, and Peroxisomal diseases. Other diseases that have been diagnosed included myopathies, ataxias, familial cholesterol and lipoprotein abnormalities, osteogenesis imperfecta, and dwarfism. The exact number of cases is unknown. The Health Ministry provides the needed resources that make possible that six children with Gaucher diseases receive enzyme replacement therapy (Fig. 4A, B). Although the hospital has a central laboratory that has the capacity to perform some biochemical genetics studies such as urine organic acids, urine glycosaminoglycans and reducing substances, the vast majority of the testing needs to be sent out overseas. The Maternity of Nuestra Señora de la Altagracia, a public service maternity, has a cytogenetic laboratory that performs conventional chromosome analysis and is staffed by two cytogeneticists.

Private health centers in the DR do not offer genetics as a clinical service. There are many private laboratories that offer most genetic testing available such as gene sequencing, microarray, whole exome sequencing, enzyme analysis and others, but they send the samples overseas for testing, primarily to the US. This also applies to metabolic testing as well as newborn screening for hemoglobinopathies, endocrinopathies, and inborn errors of metabolism, service that is offered through private laboratories. Cytogenetics is available in the country. There is a private cytogenetic laboratory that has been providing services for more than 30 years, Laboratorio de Citogenética Rosa Guerra. Genetic counseling is rarely offered and the only clinic that offers this service is the clinic at the RCC Children's Hospital. Prenatal testing is offered through private obstetric services for aneuploidies. Amniocentesis and chorionic villi sampling are offered, but their availability and costs makes them almost an impossible option for a large percentage of the population. Public sector prenatal care may be limited to ultrasounds and biophysical profiles depending on the availability of technology and trained staff. Follow‐up and management of patients with genetic diseases is limited. How the results of these tests are interpreted and informed to the families is also unclear.

## Family Planning and Reproductive Law

The Penal Code of 1948 (section 317) prohibits the performance of all abortions (Penal Code of the Dominican Republic 1948). Under the general principles of criminal legislation, however, abortion is permitted to save the life of the pregnant woman on grounds of necessity. Thus, in practice, both the legal and medical professions consider abortion performed to save the life of the woman to be legal in the DR (Penal Code of the Dominican Republic 1948; United Nations 2014) (see Table 4).

**Table 4:** Abortion policy in the Dominican Republic

Abortion policy – grounds on which abortion is permitted	
To save the life of the woman	Yes^a^
To preserve physical health	No
To preserve mental health	No
Rape or incest	No
Fetal impairment	No
Economic or social reason	No
Available on request	No

a The abortion law does not expressly allow abortions to be performed to save the life of the woman, but the general principles of criminal legislation allow abortions to be performed for this reason on the grounds of necessity.

Persons who perform an illegal abortion are subject to imprisonment for an unspecified term, as are women who cause their own abortions or consent to an abortion. Any person who puts a pregnant woman in contact with another person for the purpose of abortion is subject to 6 months to 2 years in prison, so long as the abortion is performed. If the person performing the abortion belongs to the medical or paramedical profession, he or she is subject to 5–20 years of hard labor or prison.

Family planning and maternal‐child health services are provided in the DR. The State provides a wide range of contraceptive methods, with assistance from several NGOs, such as the Dominican Family Welfare Association (Asociación Dominicana Pro‐Bienestar de la Familia/PROFAMILIA). PROFAMILIA also sells low‐priced contraceptives through a large network of distributors in communities around the country and subsidizes contraceptives marketed in pharmacies and stores. This assistance is complemented with information, education, and services that target adolescents in particular. Sterilization is also provided through the public sector, and the incidence of female sterilization is high, with more than 40% of women of reproductive age choosing sterilization (United Nations 2014).

The incidence of induced abortion is also high in the DR. The number of induced abortions was estimated to be about 65,000 per year, or one abortion for every three births, by the end of the 1980s and about 82,000 in the beginning of the 1990s (United Nations 2014).

Despite its illegality, abortion is performed with impunity in private hospitals and clinics, as well as in more clandestine and unsafe circumstances by the pregnant woman herself or by a midwife. The use of misoprostol by Dominican women with no medical supervision has been studied and the numbers revealed a significant number of women who are endangering their lives (Miller et al. 2005). In private hospitals and clinics, physicians generally classify an abortion as therapeutic when it is performed to save the life of the woman, but many are performed on eugenic and health grounds. So widespread is this practice that many physicians believe that abortion is legal on therapeutic grounds, and some public hospitals have gone so far as to develop procedures and regulations to consider and authorize the abortion. Generally, the procedure entails obtaining the written authorization of another medical colleague and/or placing the decision in the hands of a panel of physicians. Once an abortion is authorized, the legal authorities are notified that the abortion will be performed. In private clinic settings, a medical colleague is consulted. Sometimes, the abortion is performed only with the consent of the pregnant woman's spouse (United Nations 2014). But in theory, abortions should not be performed under any circumstances and there have been recent cases where the mother's health was in jeopardy while pregnant and the authorities did not allow the abortion. These cases get national attention, and the Catholic Church intervenes. Cases of pregnant mothers needing chemotherapy have been challenging.

## Congenital Malformations, Chromosomal Abnormalities, and Mendelian Disorders

The prevalence and incidence of congenital malformations and dysmorphic syndromes in the DR are unknown; however, The Latin‐American Collaborative Study of Congenital Malformations included the DR in 1990 (Castilla and Orioli 2004). A separate single institution registry for congenital abnormalities and Mendelian disorders was available during the 1990s, but this registry no longer exists. Currently, a national registry for genetic diseases is underway. Chromosomal abnormalities and dysmorphic syndromes have been reported in the Dominican medical literature; however, a comprehensive statistical review of these reports in not available. A small number of pediatricians and neonatologists have their personal registry. These personal registries depend on the physician's patient population which limits the statistical value of such registries. The population may not be representative and limitations such as lack of proper genetic testing interfere with diagnosis. Table 5 shows 15 years of experience of a cytogenetic laboratory in Santo Domingo from 1977 to 1991. This laboratory studied 1459 cases during this period. There were 622 males and 788 females. Forty‐nine cases had undetermined sex. Chromosomal abnormalities were detected on 513 cases. Methods for this study were not described in the report. This study represented the first statistical and long‐term experience and report about chromosomal abnormalities in the DR (Guerra Valdez et al. 1991). For the purpose of this report, we were able to contact the authors of this report to check where they are now. The laboratory continues its work and we were able to obtain an update. Table 6 shows the experience of this laboratory from 2013 to 2015. During this time, 601 genetic tests were ordered. The samples included tissues from products of conception, amniotic fluid, peripheral blood, and bone marrow. A small number of samples were submitted for diagnosis of specific diseases. Results showed various aneuploidies and chromosomal rearrangement abnormalities.

**Table 5:** Fifteen years of cytogenetics in the Dominican Republic

Demographics	
Total cases	1459
Male	622
Female	788
Undetermined sex	49
Results	
Cases with chromosomal abnormality	513 (35%)
Down syndrome	192 (37%)
Turner syndrome	93 (18%)
Intersex	48 (9%)

**Table 6:** Three years of cytogenetics in the Dominican Republic: an update from 2013 to 2015^a^

Number of cases	601 cases
Tissue culture from product of conception: 56	
Negative	27
Positive	20
No growth	9
Trisomy 16	5
Trisomy 15	3
Trisomy 13 and Trisomy 21	2 each
Trisomies 7, 14, 22	One of each
45 X	3
Amniotic fluid culture: 36	
Negative	27
Positive	9
Trisomy 21	4
Trisomies 13 and 18	One of each
46,XX,7q+	1
46,XY,t(8;20)(22q;11q)mat	1
46,XY,inv(9)(p13q13)	1
Peripheral blood: 495	
Negative	412
Positive	84
Down syndrome	21
Turner syndrome	3
Edward syndrome	1
Klinefelter syndrome	2
46, XY or XX, inversion chromosome 9	6
47,XY or XX, inversion 9 + extra chromosome	3
46,XY,inv(2) (p11q13)	1
46,XX,22p‐	1
46,XY,rob(14;21)(q10;q10)	1
46,XX,t(3;4)(p12q13)	1
Mosaicism	5
Ring chromosome	3
46 XX with male phenotype	1
46 XY with female phenotype	1
Bone Marrow: 1	
46,XX,t(9;22)(q34;q13)	1
FISH: 3	
Negative	2
Positive	1
Deletion for prader–willi syndrome	1
Samples for gene testing: 9	
Negative	8
Positive	1
Prader–Willi syndrome uniparental disomy	1
Negative samples tested for fragile X syndrome (6 cases), Neurofibromatosis type 1 (1 case) and Prader–Willi syndrome (1 case)	

a Results shown are the most significant findings. Sex of the cases was not taken into consideration for this table, but the information is available.

In the DR, as mentioned previously, there is only one clinic that provides genetic services. This clinic cannot provide these services to the entire Dominican population and resources are limited. The follow‐up and clinical services available for patients with diagnosed chromosomal abnormalities and other genetic diseases is unclear. It is safe to assume that their pediatricians provide routine services for those affected with a genetic disease, but genetic counseling and family planning resources are available to a few and how these services are provided is unclear. Patients, who have the economic resources to do so, travel to other countries for evaluations by specialists that are not available in the DR.

## Newborn Screening and Inborn Errors of Metabolism

The National Office of Statistics reported that 339,000 children and adolescents were living with some form of disability in the DR during 2013 (Oficina Nacional de Estadistica 2013). The report does not distinguish between disabilities caused by an accident or a medical condition. Among children with disabilities, causes such as inborn errors of metabolism have not been accounted for. As with chromosomal abnormalities, a registry for inborn errors of metabolism and rare diseases does not exist. Newborn screening (NBS) in Latin America took its first steps in the mid‐1970s. Borrajo (2007) reported the status of NBS in Latin America in the 21st century in 2007. In this report, while countries such as Cuba and Costa Rica were described as having optimal NBS programs, the DR was described as having minimal isolated and nonorganized NBS activity. This activity was mainly performed by private health care institutions that offered NBS to their patients and these tests were performed upon the patient's agreement and ability to pay for such test. Currently, there are two private institutions that offer an expanded NBS panel to patients who have health coverage or can pay a fee for the service. These private institutions take the NBS sample at the baby's place of birth and send it to the USA for testing at one of the many NBS programs laboratories. One of these private institutions had 30 cases with an abnormal NBS from 2013 to 2015. The total number of screenings performed during this period was not available. Fourteen cases were presumptive for glucose‐6‐phosphate dehydrogenase deficiency, seven cases for sickle cell trait, two for hemoglobin C trait, and two for Krabbe disease. Congenital hypothyroidism, Niemann–Pick A/B, Cystic Fibrosis, phenylketonuria, and high methionine had one case each. It is estimated that 0.9% of all Dominican babies born annually are screened this way.

In 2013, the first lady of the DR opened the country's largest center for the awareness and management of disabilities, Centro de Atencion Integral para la Discapacidad (CAID), as an answer to the limited resources available for children with disabilities. Pediatricians and other healthcare workers involved in the project agreed that this was a much needed and overdue project, but also advocated for the creation of a NBS program that would identify infants with reversible causes of intellectual disabilities. A bill for the creation of the national NBS program was presented to the Dominican congress and on December 7th, 2015, President Danilo Medina signed the law that created the National Newborn Screening and High Risk program (National Newborn Screening and High Risk Program 2015). The program will evolve in three stages. The first stage will consist in the screening of newborns of three maternities in Santo Domingo, maternities that register the most births in the DR. The second stage will include other centers from the different health regions of the country selected according to the population density and health risks of the region. The third stage will extend the NBS program nationwide and is expected that at least 95% of all newborns will be screened through the national NBS program. The program also includes the creation of a NBS laboratory in Santo Domingo.

A National Newborn Screening Counsel was created for the execution of the NBS program and it is composed of the public health minister, the first lady of the Dominican Republic, the director of the national NBS program, the president of the national council for childhood and adolescence, the president of the national council for disabilities, and the president of the Dominican Pediatric Society. During its first stage, the NBS program will screen for five conditions: sickle cell disease and other hemoglobinopathies, congenital hypothyroidism, phenylketonuria and other disorders of phenylalanine metabolism, galactosemia, and glucose‐6‐phosphate dehydrogenase deficiency. Samples for the NBS program shall be obtained 48 h after birth and up to 28 days after birth. The NBS program must guarantee timely results and provide the adequate treatment, follow‐up and counseling for the affected baby and family (National Newborn Screening and High Risk Program 2015).

With the implementation of the NBS program, the national database for metabolic diseases and related conditions was also created. This is a much needed database since until now; the statistics regarding genetic diseases in the DR is poor and incomplete. The pilot studies as well as the construction of the NBS laboratory are currently underway (National Newborn Screening and High Risk Program 2015).

## Cancer Genetics

Data from the National Oncology Institute showed that 2017 Dominicans were diagnosed with a new malignancy in 2008. The most common malignancies affecting Dominicans during 2008 were breast cancer, cervical cancer, and head and neck cancers. Molecular pathology and cancer genetics services are not available throughout the country. Most of the services available to the population are within the private healthcare sector (Pan American Health Organization 2012). Cruz‐Correa et al. (2015)studied a cohort of patients which included Dominicans looking for germline mutations in *MLH1, MSH2*, and *MSH6* genes that cause Lynch syndrome, but this research was performed in Puerto Rico (Thigpen et al. 1992).

## Rare Diseases and Medical Research in the Dominican Republic

Medical research has been present in the DR since medical training started in the island. Medical school and other healthcare related professions' programs combine teaching with training early enough in medical and health educations. The DR is not leading the world in medical research and innovation; however, there are academic and professional institutions that with the correct guidance and resources are capable of performing world class research. Medical school graduates with interest in medical research look for opportunities in other countries, but only a handful return to the DR. The various professional institutions in the DR that supervise medical training and excellence such as the Dominican Medical College, Dominican Society of Pediatrics, and the National Counsel for Bioethics and Health (CONABIOS) are also responsible for promoting medical research and continuing medical education. Independent Review Boards (IRBs) are part of these organizations and are also part of the structure of large hospitals in the DR that have affiliations with medical schools. However, regulation of clinical research with human subjects is still disappointing and national legislature covering research does not exist. The current guidelines for research regulation are confusing and vague. Research projects in the DR are sponsored by many of these institutions and international collaboration also exists. Research articles in genetics that make reference to the DR or the Dominican population are not many, but have contributed with new knowledge (see Table 7). The CONABIOS is the government agency for medical research regulation in the DR that includes human subjects as research participants. This agency should also monitor and regulate research projects sponsored by private institutions and also those projects with international collaboration. In their website, http://conabios.gob.do/, similar to clinicaltrials.gov, the CONABIOS has the title for 150 research projects that are presumed active in the DR from 2013 to 2016. Eight of these projects were in the area of genetics. Cancer genetics, genome studies, and inborn errors of metabolism were the fields being studied. Two studies are worth mentioning; the first is the sequencing of the Dominican genome sponsored by the National Geographic Society in collaboration with the Dominican Archeological Society. The second study is a multicenter trial, headed in the DR by Dr Ceila Perez‐Estrella de Ferrán from the RCC Children's Hospital which performs evaluations in order to quantify the changes in height and weight in children with Hunter syndrome that are receiving Elaprase therapy. This study is open and recruiting patients. It is being sponsored by Shire Human Genetics Therapies.

**Table 7:** The Dominican Republic in the genetics literature (Cai et al. 1996; Castilla and Orioli 2004; Della‐Morte et al. 2010, 2011; Garcia‐Godoy 1980; Garone et al. 2011; Goicochea de Jorge et al. 2002; Hamaguchi et al. 2004; Hickey et al. 2014; Imperato‐McGinley et al. 1974; Iyer et al. 2014; Jimenez 1978; Lee et al. 2014; Mendoza 1997; Ministry of Health 2014; Romas et al. 2002; Rosenberg et al. 1978; Rosenthal et al. 2014; Schiessl‐Weyer et al. 2015; Thigpen et al. 1992; Vardarajan et al. 2015; Wang et al. 2012; Zatkova 2011)

Author	Title	Year
Imperato‐McGinley J	Steroid 5alpha‐reductase deficiency in man: an inherited form of male pseudohermaphroditism	1974
Henrietta Kotlus Rosenberg	The Dominican Republic Conjoined Twins: Ischiopagus, Tetrapus, Omphalopagus	1978
Fidelio A. Jimenez	The First Autopsy in the New World	1978
Franklin Garcia‐Godoy	Cleft lip and palate in Santo Domingo	1980
Imperato‐McGinley J	The prevalence of 5 alpha‐reductase deficiency in children with ambiguous genitalia in the Dominican Republic	1986
Anice E. Thigpen	Brief Report: The molecular basis of steroid 5 alpha‐reductase deficiency in a large Dominican kindred	1992
Li‐Qun Cai	5 Alpha‐Reductase‐2 gene mutations in the Dominican Republic	1996
Hugo R. Mendoza	A newly recognized autosomal dominant ectodermal dysplasia syndrome: the odonto‐tricho‐ungual‐digital‐palmar syndrome	1997
E Goicoechea de Jorge	Alkaptonuria in the Dominican Republic: identification of the founder AKU mutation and further evidence of mutation hot spots in the HGO gene	2002
Stavra N. Romas	Familial Alzheimer disease among Caribbean Hispanics: a reexamination of its association with APOE	2002
Eduardo Castilla	ECLAMC: The Latin‐American collaborative study of congenital malformations	2004
Kazuyuki Hamaguchi	The PC‐1 Q121 Allele Is Exceptionally Prevalent in the Dominican Republic and Is Associated with Type 2 Diabetes	2004
David Della‐Morte	Genetic linkage of serum homocysteine in Dominican families: the family study of stroke risk and carotid atherosclerosis	2010
David Della‐Morte	A follow‐up study for left ventricular mass on chromosome 12p11 identifies potential candidate genes	2011
Caterina Garone	Clinical and genetic spectrum of mitochondrial neurogastrointestinal encephalomyopathy	2011
Andrea Zatkova	An update on molecular genetics of Alkaptonuria (AKU)	2011
Liyong Wang	Fine Mapping Study Reveals Novel Candidate Genes for Carotid Intima‐Media Thickness in Dominican Republican Families	2012
Shoba Iyer	Significant interactions between maternal PAH exposure and haplotypes in candidate genes on B[a]P‐DNA adducts in a NYC cohort of non‐smoking African‐American and Dominican mothers and newborns	2013
Jorge Rosenthal	Neural tube defects in Latin America and the impact of fortification: a literature review	2014
Kathleen T. Hickey	Cardiac genetic testing: a single‐center pilot study of a Dominican population	2014
Joseph H. Lee	Disease‐related mutations among Caribbean Hispanics with familial dementia	2014
Marcia Cruz Correa	Clinical characterization and mutation spectrum in Caribbean Hispanic families with Lynch syndrome	2015
Jasmin Schiessl‐Weyer	Acanthocytosis and the c.680 A>G Mutation in the PANK2 Gene: A Study enrolling a cohort of PKAN Patients from the Dominican Republic	2015
Badri N. Vardarajan	Coding Mutations in SORL1 and Alzheimer disease	2015

## Medical Genetics Training

In 2008, the Dominican physician association had 20,000 members, 40% were registered as specialists, but no clinical geneticist or metabolic geneticist was registered with the association (Pan American Health Organization 2012). There are 17 institutes of higher learning in the country that offer degrees in health programs for all levels: technician, graduate, and postgraduate. In medical school, clinical genetics is offered as a subject and during the clinical rotation years, a large percentage of the medical students who are interested in genetics are encouraged to do their genetics rotation at the genetic clinic in RRC Children's Hospital. Specialty training programs are available throughout the DR. Pediatrics is offered through eight programs and training for 11 pediatric subspecialties is also offered. Internal Medicine and Family Medicine are offered through 14 and 17 programs, respectively. Nine programs offer training in Obstetrics and Gynecology (Ministry of Health 2014). These are the specialists that will handle any genetic case in the DR with little to no training in genetics. Within the Ministry of Health, The Ministry of Higher Education and the private universities in the DR, there is no genetic training program offered, masters or doctorate. The staff members who work at the cytogenetics laboratory under the Ministry of Health were trained in Cuba and one of the genetics professors at a local medical school has an online‐degree in forensic genetics from a university in Spain. Training programs for genetic counseling and other fields in genetics are also needed in the country.

## Opportunities in Genomic Medicine in the Dominican Republic

The DR offers numerous opportunities in medical research. Not only in the fields of genetics, but also in other science fields that are yet to reach their potential. The number of medical institutions is growing and screening programs are being implemented in the country. With the implementation of screening programs as well as disease registries by the Dominican government, patients can now be organized and different cohorts for an endless amount of research projects will be available. The concept of medical research is known and understood in the DR, but the limited resources and absence of trained scientists in the different areas of medical research is currently stopping progress in this area. With the results of the Dominican genome and nationwide implantation of the newborn screening programs, samples will be collected and can be made available for researchers who are interested in doing population genetics studies in the DR. For example, the field of epigenetics has not been studied in the DR. Cancer genetics and rare diseases programs are needed and the opportunities for collaborations with Dominican researchers must be considered. Therapies, devices, and medical foods are not ubiquitously available in the DR; introduction of these into the Dominican health care system for the management of genetic diseases will open the doors for many established research and educational groups to come and bring their expertise.

## Conclusions and Thoughts

The Dominican Republic's healthcare system needs improvements in many areas. It is true that there are other basic health care issues affecting the DR, but children and adults with genetic diseases are often undiagnosed, cannot receive adequate treatment, and many feel neglected and abandoned. Education in genetic diseases is required in all spheres of the population. Patients with malformations, dysmorphic syndromes, albinism, and other diseases are often the object of discrimination and bullying. This also happens to people with any disability in general. In the rural areas of the country, witchcraft or the “devil's hand” is sometimes thought to be the cause for these medical conditions resulting in babies being abandoned in hospitals and children and adults with these issues being isolated from the community. The psychological harm that these patients endure is endless and education for the population is needed in order to stop this behavior. Medical professionals need educations as well. Genetics is included in the programs of many medical schools, but postgraduate training is not available. Training in genetic counseling, molecular, biochemical, and cytogenetics genetics is not available either. Genetic counseling is given by pediatricians, obstetricians, and oncologist in limited areas and the follow‐up for patients with genetic diseases is complicated or uncertain in many cases. The implementation of training programs will be a partial solution to this need, as it will take many years to train enough healthcare workers to fulfill the demand. At this point, collaborations with international groups and the use of telemedicine for consultation will be the best way for local physicians to obtain answers, though this should not be considered a permanent solution. Besides this, medical statistics programs need improvement. The current statistics for the country come from different sources and do not cover the same indicators and populations. This creates confusion and allocation of needed resources may not be achieved satisfactorily. The statistics programs should be one. If a country does not have reliable statistics, it does not know its achievements and cannot identify its needs. Lastly, a uniform legislature for clinical research regulation is needed in order to build the necessary foundation that will encourage the young medical generation of the DR to perform and promote clinical research.

## Websites

Agency, C. I. A. 2013. The Dominican Republic. CIA world facts book. https://www.cia.gov/library/publications/the-world-factbook/geos/dr.html

Ministry of Health of the Dominican Republic: http://www.sespas.gov.do/.

Ministry of Health: Demographic and Health Survey: Dominican Republic 2013. http://countryoffice.unfpa.org/dominicanrepublic/drive/DRDHS2013-Final02-10-2013.pdf.

National Counsel for Bioethics in Health: http://conabios.gob.do/.

National Statistics Office of the Dominican Republic (Oficina Nacional de Estadística – ONE) http://www.one.gob.do/#.

Residency Training Programs in the Dominican Republic: http://www.msp.gob.do/oai/documentos/Requisitos/REQU_ConcursoMedico2014.pdf.

The World Bank: http://data.worldbank.org/country/dominican-republic.

World Health Organization: Dominican Republic; http://www.who.int/countries/dom/en/.

## Conflict of Interest

None declared.

## References

[mgg3224-bib-0002] Borrajo, G. 2007 Newborn Screening in Latinamerica at the beginning of the 21st century. J. Inherit. Metab. Dis. 30:466–481. 17701285 10.1007/s10545-007-0669-9

[mgg3224-bib-0003] Cai, L.‐Q. , Y.‐S. Zhu , M. Katz , et al. 1996 5‐Alpha reductase‐2 gene mutations in the Dominican Republic. J. Clin. Endocrinol. Metab. 81:1730–1735. 8626825 10.1210/jcem.81.5.8626825

[mgg3224-bib-0004] Castilla, E. , and I. Orioli . 2004 ECLAMC: the Latinamerican collaborative study of congenital malformations. Community Genet. 7:76–94. 15539822 10.1159/000080776

[mgg3224-bib-0005] Cruz‐Correa, M. , Y. Diaz‐Algorri , J. Perez‐Mayoral , et al. 2015 Clinical characterization and mutation spectrum in Caribbean Hispanic families with Lynch syndrome. Fam. Cancer 14:415–425. 25782445 10.1007/s10689-015-9795-y PMC4560599

[mgg3224-bib-0006] Della‐Morte, D. , A. Beecham , T. Rundek , et al. 2010 Genetic linkage of serum homocysteine in Dominican families. Stroke 41:1356–1362. 20489178 10.1161/STROKEAHA.109.573626 PMC2914470

[mgg3224-bib-0007] Della‐Morte, D. , A. Beecham , T. Rundek , et al. 2011 A follow‐up study for left ventricular mass on chromosome 12p11 identifies potential candidate genes. BMC Med. Genet. 12:100. 21791083 10.1186/1471-2350-12-100 PMC3199748

[mgg3224-bib-0008] Garcia‐Godoy, F. 1980 Cleft lip and cleft palate in Santo Domingo. Community Dent. Oral Epidemiol. 8:89–91. 6934063 10.1111/j.1600-0528.1980.tb01263.x

[mgg3224-bib-0009] Garone, C. , S. Tadesse , and M. Hirano . 2011 Clinical and genetic spectrum of mitochondrial neurogastrointestinal encephalomyopathy. Brain 134: 3326–3332. 21933806 10.1093/brain/awr245 PMC3212717

[mgg3224-bib-0010] Goicochea de Jorge, E. , I. Lorda , M. Gallardo , et al. 2002 Alkaptonuria in the Dominican Republic: identification of a founder AKU mutation and further evidence of mutation hotspots in the HGO gene. J. Med. Genet. 39:e40. 12114497 10.1136/jmg.39.7.e40 PMC1735184

[mgg3224-bib-0011] Guerra Valdez, R. , M. Jacquez Gutierrez , and Z. Herrera Ramirez . 1991 Fifteen Years of Cytogenetics in the Dominican Republic. Laboratory of Genetics at the Gomez Patiño Clinic, Santo Domingo, Dominican Republic.

[mgg3224-bib-0012] Hamaguchi, K. , H. Terao , Y. Kusuda , et al. 2004 The PC‐1 Q121 allele is exceptionally prevalent in the Dominican Republic and is associated with type 2 diabetes. J. Clin. Endocrinol. Metab. 89:1359–1364. 15001634 10.1210/jc.2003-031387

[mgg3224-bib-0013] Hickey, K. , J. Taylor , R. Sciacca , et al. 2014 Cardiac genetic testing: a single‐center pilot study of a Dominican population. Hisp. Health Care Int. 12:183–188. 25521782 10.1891/1540-4153.12.4.183 PMC7817112

[mgg3224-bib-0014] Imperato‐McGinley, J. , L. Guerrero , T. Gautier , and R. E. Peterson . 1974 Steroid 5alpha‐reductase deficiency in man: an inherited form of male pseudohermaphroditism. Science 186:1213–1215. 4432067 10.1126/science.186.4170.1213

[mgg3224-bib-0015] Iyer, S. , F. Perera , B. Zhang , et al. 2014 Significant interactions between maternal PAH exposure and haplotypes in candidate genes on B(a)P‐DNA adducts in a NYC cohort of non‐smoking African‐American and Dominican mothers and newborns. Carcinogenesis 35: 69–75. 24177223 10.1093/carcin/bgt339 PMC3871941

[mgg3224-bib-0016] Jimenez, F. 1978 The first autopsy in the New World. Bull. N.Y. Acad. Med. 54:618–619. 350323 PMC1807498

[mgg3224-bib-0017] Lee, J. , A. Kahn , R. Cheng , et al. 2014 Disease‐related mutations among Caribbean Hispanics with familial dementia. Mol. Genet. Genomic Med. 2:430–437. 25333068 10.1002/mgg3.85 PMC4190878

[mgg3224-bib-0018] Mendoza, H. 1997 A newly recognized autosomal dominant ectodermal dysplasia syndrome: The odonto‐tricho‐ungual‐digital‐palmar syndrome. Am. J. Med. Genet. 71:144–149. 9217212 10.1002/(sici)1096-8628(19970808)71:2<144::aid-ajmg5>3.0.co;2-y

[mgg3224-bib-0019] Miller, S. , T. Lehman , M. Campbell , et al. 2005 Misoprostol and declining abortion‐related morbidity in Santo Domingo, Dominican Republic: a temporal association. BJOG 112:1291–1296. 16101610 10.1111/j.1471-0528.2005.00704.x

[mgg3224-bib-0020] Ministerio de Salud de la Republica Dominicana . 2001 Ley General de Salud: Ley No. 42‐01, Santo Domingo, Dominican Republic.

[mgg3224-bib-0021] Ministerio de Salud Publica de la Republica Dominicana . 2007 Direccion de Information y Estadistica de Salud.

[mgg3224-bib-0022] Ministry of Health . 2014 Residency Training Programs in the Dominican Republic: Academic Year 2014. Santo Domingo, Dominican Republic.

[mgg3224-bib-0023] Montinaro, F. , et al. 2015 Unravelling the hidden ancestry of American admixed populations. Nat. Commun.. See Supplementary Data. Doi:10.1038/ncomms7596 10.1038/ncomms7596 PMC4374169 25803618

[mgg3224-bib-0024] National Newborn Screening and High Risk Program . 2015 Decree No. 380‐15, Presidency of the Dominican Republic: national newborn screening and high risk program.

[mgg3224-bib-0025] Oficina Nacional de Estadistica (ONE) . 2007‐2012. Indicadores de Recursos Humanos para el Sector Publico de la Republica Dominicana.

[mgg3224-bib-0026] Oficina Nacional de Estadistica . 2008‐2013a. Indicadores de Salud para el Sector Publico de la Republica Dominicana.

[mgg3224-bib-0027] Oficina Nacional de Estadistica (ONE) . 2008‐2013b. Mortalidad Materna en la Republica Dominicana.

[mgg3224-bib-0028] Oficina Nacional de Estadistica (ONE) , 2010a Censo 2010, Volumen III: Caracteristicas Demograficas Basicas de la Republica Dominicana.

[mgg3224-bib-0029] Oficina Nacional de Estadistica (ONE) , 2010b Censo 2010, Volumen V: Caracteristicas Economicas de la Republica Dominicana.

[mgg3224-bib-0030] Oficina Nacional de Estadistica (ONE) , 2010c Censo 2010, Volumen VI: Migracion, Fecundidada, Mortalidad para la Republica Dominicana.

[mgg3224-bib-0031] Oficina Nacional de Estadistica (ONE) . 2013 Poblacion con Alguna Discapacidad en la Republica Dominicana.

[mgg3224-bib-0032] Pan American Health Organization . 2012 Health in the Americas. Edition: Country, Dominican Republic.

[mgg3224-bib-0033] Mir, P. 1949 “There is a country in the world” Poem.

[mgg3224-bib-0034] Penal Code of the Dominican Republic . 1948 Section 317.

[mgg3224-bib-0035] Romas, S. , V. Santana , J. Williamson , et al. 2002 Familial Alzheimer disease among Caribbean Hispanics: a reexamination of its association with APOE. Arch. Neurol. 59:87–91. 11790235 10.1001/archneur.59.1.87

[mgg3224-bib-0036] Rosenberg, H. , T. Spackman , A. Chait , et al. 1978 The Dominican Republic Conjoined Twins: Ischiopagus, Tetrapus, Omphalopagus. Am. J. Roentgenol. 130:921–926. 417592 10.2214/ajr.130.5.921

[mgg3224-bib-0037] Rosenthal, J. , J. Casas , D. Taren , et al. 2014 Neural tube defects in latinamerica and the impact of fortification: a literature review. Public Health Nutr. 17:537–550. 23464652 10.1017/S1368980013000256 PMC4479156

[mgg3224-bib-0038] Schiessl‐Weyer, J. , P. Roa , F. Laccone , et al. 2015 Acanthocytosis and the c.680 A>G mutation in the PANK2 gene: a study enrolling a cohort of PKAN patients from the Dominican Republic. PLoS ONE 10:e0125861. 25915509 10.1371/journal.pone.0125861 PMC4411072

[mgg3224-bib-0039] The World Bank . 2014 Dominican Republic, Available at: data.worldbank.org.

[mgg3224-bib-0040] Thigpen, A. , D. Davis , T. Gautier , et al. 1992 Brief Report: the molecular basis of steroid 5‐alpha reductase deficiency in a large dominican kindred. N. Engl. J. Med. 327:1216–1219. 1406794 10.1056/NEJM199210223271706

[mgg3224-bib-0041] United Nations, Department of Economic and Social Affairs, Population Division . 2014 Abortion Policies and Reproductive Health around the World (United Nations publication, Sales No. E.14.XIII.11).

[mgg3224-bib-0042] Vardarajan, B. , Y. Zhang , J. Lee , et al. 2015 Coding mutations in SORL1 and Alzheimer disease. Ann. Neurol. 77:215–227. 25382023 10.1002/ana.24305 PMC4367199

[mgg3224-bib-0043] Wang, L. , A. Beecham , D. Zhuo , et al. 2012 Fine mapping study reveals novel candidate genes for carotid intima‐media thickness in Dominican Families. Circ. Cardiovasc. Genet. 5(2):234–241. 22423143 10.1161/CIRCGENETICS.111.961763 PMC3341091

[mgg3224-bib-0044] World Health Organization . 2013 Health Indicators for the Dominican Republic 2013.

[mgg3224-bib-0045] Zatkova, A . 2011 An update on molecular genetics of Alkaptonuria. J. Inherit. Metab. Dis. 34(6):1127–1136. 21720873 10.1007/s10545-011-9363-z

